# No practice and still perfect: Different routes for promoting offline gains in perceptual learning

**DOI:** 10.1016/j.isci.2026.117839

**Published:** 2026-10-13

**Authors:** Ylka Kolken, Mark J. Roberts, Parisa Naseri, Federico De Martino, Nitzan Censor, Peter De Weerd

**Affiliations:** 1 Department of Cognitive Neuroscience, Maastricht University, Oxfordlaan 55, 6229EV Maastricht, the Netherlands; 2 School of Psychological Sciences and Sagol School of Neuroscience, Tel Aviv Univeristy, Tel Aviv 69978, Israel

**Keywords:** attention, consolidation, perceptual learning, texture discrimination task

## Abstract

Visual perceptual learning is thought to require extensive training, but a few reminder trials can sometimes replace daily practice. In a 5-day texture discrimination learning experiment, we showed that both accounts need amendment. Following an initial training session, we found that further skill acquisition can be accomplished either with repeated daily administration of a few reminder trials or by offline learning without any further training, depending on attentional context and task demands. In dual-task conditions involving the simultaneous discrimination of a peripheral texture target and a central fixation target, reminders were as effective as daily training, whereas no learning occurred without reminders. In contrast, in a single-task condition without a fixation task, learning emerged without reminders or training. Hence, attentional context and task demands may determine the ability of reminders or protracted consolidation to yield offline learning gains that are comparable to those delivered by daily training.

## Highlights

• Offline perceptual learning can continue for several days after a single session
• Attentional context determines how much practice is needed for learning
• Brief reminders can substitute for daily training under specific conditions

## Graphical abstract

**Figure:**
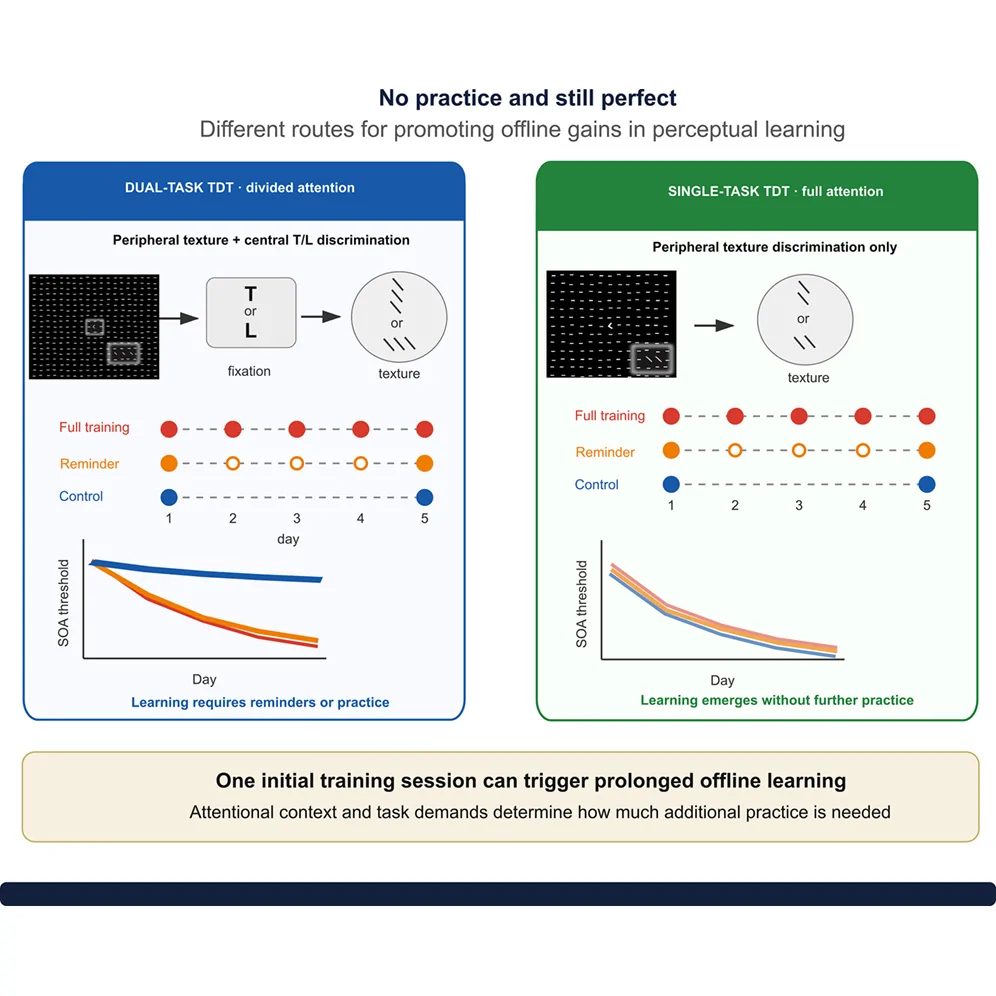

## Teaser

Neuroscience; Cognitive neuroscience; Psychology

## Introduction

Visual perceptual learning (VPL) refers to the enhancement of perceptual abilities through experience and training. As early as the nineteenth century, James^1^ and Stratton^2^ emphasized the importance of extensive daily practice in acquiring habits and refining skills. Karni and Sagi^3^ aptly captured this view in one of their hallmark papers titled “Where practice makes perfect […]”. Numerous studies have supported the idea that visual skills develop over weeks or months of deliberate daily practice.^3^^,^^4^^,^^5^^,^^6^^,^^7^^,^^8^^,^^9^^,^^10^^,^^11^^,^^12^ Contrary to this view, a recent series of studies has demonstrated that brief memory reminders produce performance gains comparable to those achieved through extensive daily training, questioning the necessity of intensive daily practice.^13^^,^^14^^,^^15^ Using a texture discrimination task (TDT), these studies demonstrated that after an initial training session, only five daily near-threshold trials (reminders) on subsequent days were sufficient to match the performance gains seen with daily practice. Amar-Halpert et al.^13^ proposed that reminder-induced reconsolidation cycles^16^^,^^17^^,^^18^^,^^19^ enable efficient perceptual learning, making extensive daily training redundant. Related work has suggested that similar reminder-based training may also induce learning benefits in other domains, including the acquisition of arbitrary numerical facts^20^ and motor skill learning.^21^ Here, we investigated the context in which reminders enable visual learning and hence permit reducing training effort without compromising learning.

The TDT paradigm used by Amar-Halpert et al.^13^ and others^14^^,^^15^^,^^22^^,^^23^^,^^24^^,^^25^ was originally devised by Karni and Sagi.^3^ It is a dual task (henceforth referred to as “dual-task TDT”) in which participants fixate and discriminate centrally presented T/L targets while also judging the orientation (horizontal vs. vertical) of a peripheral texture-defined target. Task difficulty of the dual-task TDT is manipulated via the stimulus onset asynchrony (SOA) between the texture stimulus and a following mask, and performance is measured as the SOA threshold required to achieve a predetermined level of accuracy. The T/L task is included to ensure fixation without the need for eye-tracking equipment. Because the dual-task TDT requires simultaneous central and peripheral processing, its difficulty may partly stem from dividing attention between two relevant locations. The idea that dividing attention in dual-task conditions incurs a cost for one or both tasks is well documented (for a review see work by Jans et al.^26^). For example, Kastner et al.^27^ showed that when demanding central T/L and peripheral target discrimination tasks were performed simultaneously, peripheral performance dropped from 86% to 45% correct. In the context of VPL, studies that have explicitly manipulated attention have reported reduced learning under divided attention compared to full attention.^25^^,^^28^ We therefore hypothesized that reminder benefits as studied in Amar-Halpert et al.^13^ are enabled by suboptimal learning due to limited attentional resources. In other words, reminders may only introduce benefits when previous learning was limited by divided attention due to the dual task. We addressed this question by testing the effectiveness of reminder-based learning under dual-task and single-task conditions.

An alternative account for learning in the dual-task TDT, and potentially for differences in learning among conditions, is related to anticipatory gaze positioning prior to stimulus presentation. Because attention and eye movements are closely linked,^29^^,^^30^ participants learning the task may unwittingly fixate their gaze closer to the anticipated peripheral target location, adopting a “compromise location” to optimize performance in both tasks. The possibility that gaze biases may contribute to performance and learning so far has not been tested in the dual-task TDT, because the T/L task at fixation has been considered a sufficient guarantee for accurate fixation, rendering the monitoring of eye position unnecessary. We addressed the possibility of an anticipatory gaze bias by recording participants’ eye position in the dual-task TDT. In the single-task TDT, we limited the possibility of gaze biases by restricting eye movements to a small window surrounding the fixation target.

In summary, to examine whether training and reminder effectiveness depend on attentional context, we compared learning in the standard TDT (dual-task TDT) with learning in a version omitting the fixation task (“single-task TDT”). Here, we use the term “attentional context” to refer to the task configuration that determines how many spatial locations must be selected and attended: in the dual-task TDT, participants must process information at both a central and a peripheral location, whereas in the single-task TDT only the peripheral target is behaviorally relevant. In both versions of the TDT, participants were assigned to one of three conditions: training over 5 consecutive days (*full training* condition); training only on days 1 and 5 plus 5 near-threshold reminders on days 2–4 (*reminder* condition); training only on days 1 and 5 without reminders (*control* condition). We predicted that in the dual-task TDT, the full training and reminder conditions would show similarly substantial learning between days 1 and 5, whereas the control condition would show limited learning. In the single-task TDT, without a demanding fixation task, we expected that reminders would lose their effectiveness, and that only the full training condition would produce substantial performance improvements. The learning data in the dual-task TDT was in line with our predictions, but the single-task TDT revealed a pattern of data questioning the relevance of daily training in an even more fundamental manner than previously reported.^13^ Finally, our eye movement data did not support the possibility that strategic gaze biases contributed to behavioral performance in the TDT.

## Results

To test whether the effectiveness of reminder-based learning depends on attentional context and task demands during initial encoding, we compared learning across 5 days in dual-task and single-task versions of the TDT (Figure 1). In both task versions, participants were assigned to one of three conditions: a full training condition (daily practice for 5 days), a reminder condition (initial training on day 1 followed by brief reminder trials on days 2–4), or a control condition (no practice between days 1 and 5) (Figure 2). Prior to the first session, participants were given a small number of example trials (including pre-training trials at the highest SOA and/or familiarization trials across the range of SOAs; see details in STAR Methods). Learning was quantified as the change in SOA threshold (81.6% correct) from day 1 to day 5. First, we report the results obtained in the dual-task TDT, which replicate prior work on reminder-induced learning. Second, we report performance in the single-task TDT both with the same stimulus as used in the dual TDT and with a modified stimulus, which revealed prolonged offline learning even in the absence of reminders. Finally, we address whether learning was mediated by strategic shifts in fixation.

**Figure 1:**
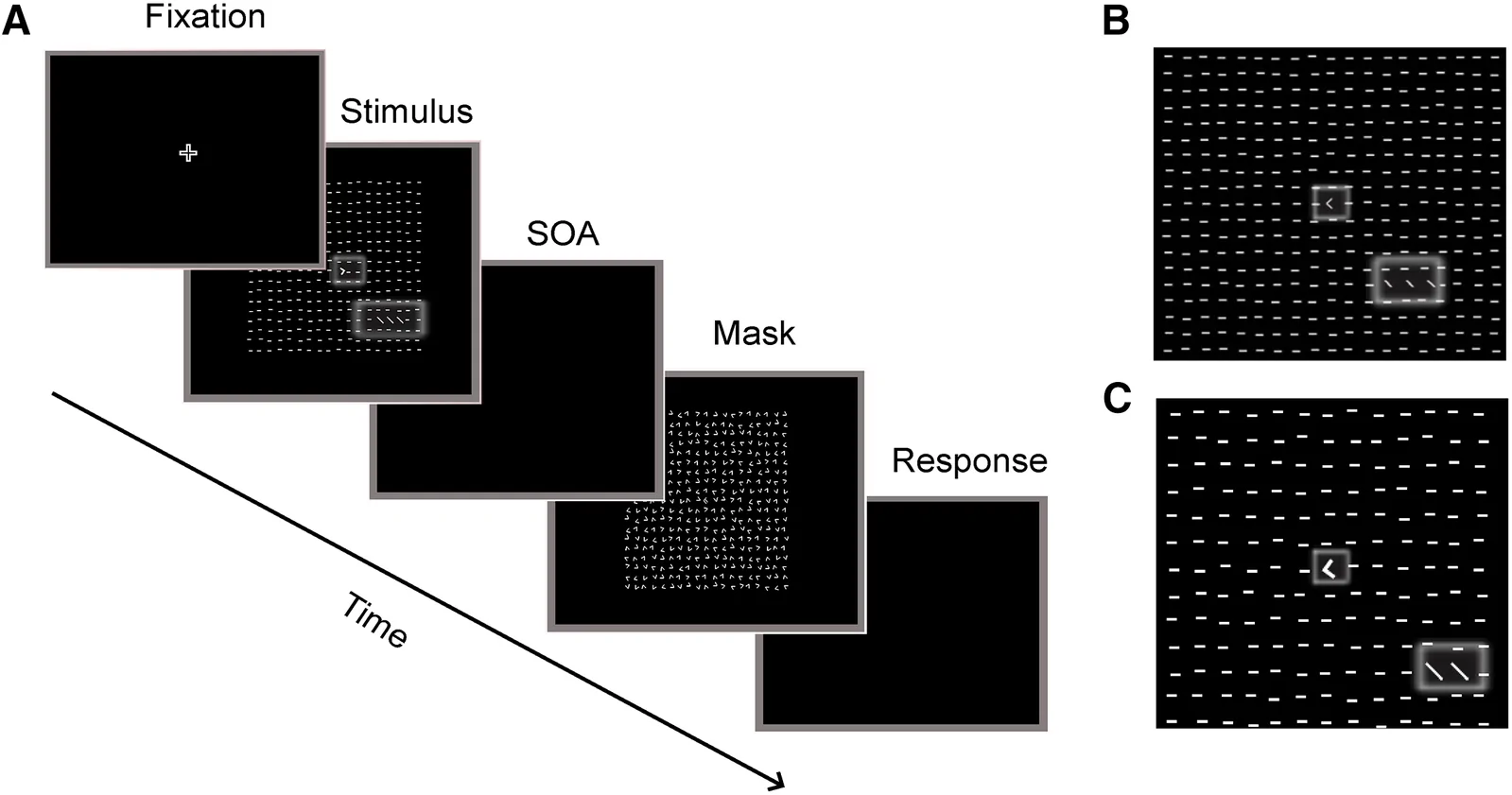
Conditions used in the two experiments (A) Trial sequence showing successive stimulus and mask presentation separated by the stimulus onset asynchrony (SOA). The white boxes inserted in the target frame indicate the location of T/L at fixation and the texture target in the periphery. These boxes are for illustration and were not shown to the participant. Participants used key presses to respond to fixation and peripheral targets, respectively. Auditory feedback was given following an incorrect response to a fixation target; no feedback was provided after responding to the peripheral texture target. (B) Stimulus used in the dual-task TDT (enlarged from the stimulus frame in A). (C) Modified stimulus (wider texture spacing and two target elements instead of three) used to increase difficulty in the single-task TDT. The central T or L character remained present in the texture, but participants were asked only to discriminate the orientation of the peripheral texture target.

**Figure 2:**
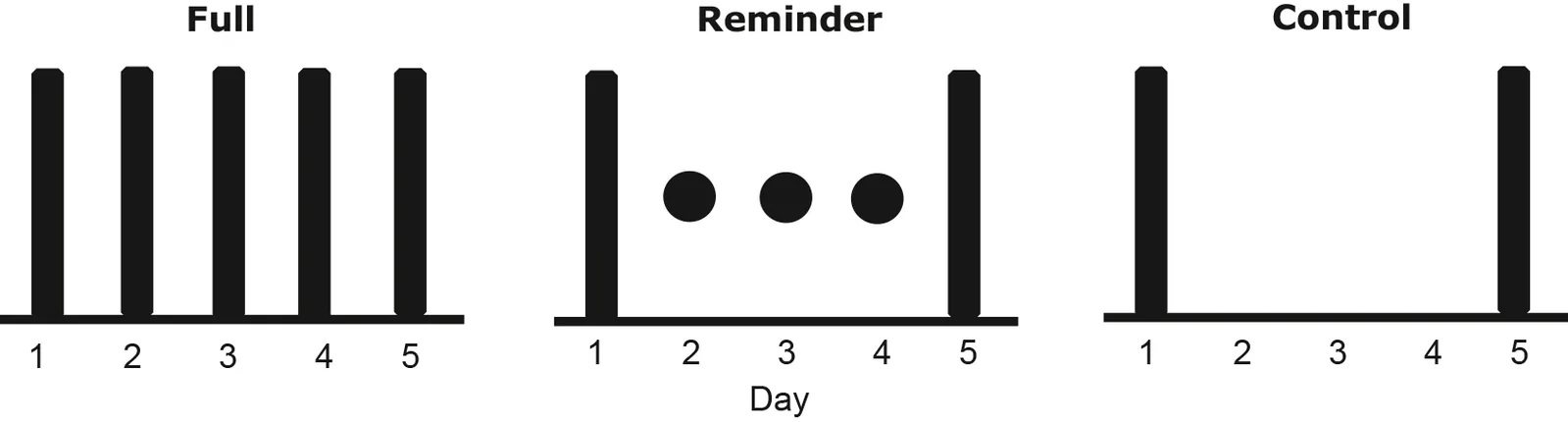
Conditions used in the two experiments The three conditions used in all experiments: full training, reminder, and control. Stripes indicate a full training session of 252 trials. Dots indicate reminder sessions of 5 trials.

### Dual-task TDT

We collected 30 complete dual-task TDT datasets, divided equally across the full training, reminder, and control conditions. The learning curves (Figure 3A) showed no threshold differences on day 1 between participants in the three conditions (F(2,54) = 0.094, *p* = 0.910). Figure 3A shows a strong reduction in SOA in the full training and reminder conditions but not the control condition. Accordingly, the linear mixed-effects model (LMM) showed a significant time × condition interaction for full training versus control (*β* = −77.120, SE = 31.27, *p* = 0.014), and reminder versus control (*β* = −67.827, SE = 31.27, *p* = 0.03), but not between full training and reminder (*β* = 9.292, SE = 31.27, *p* = 0.766) (Figure 3A, for single-subject day 1 versus day 5 thresholds see Figure 3B) condition. These results are in line with the reminder trials being as effective as full training (for all LMM effects see Table S2).

**Figure 3:**
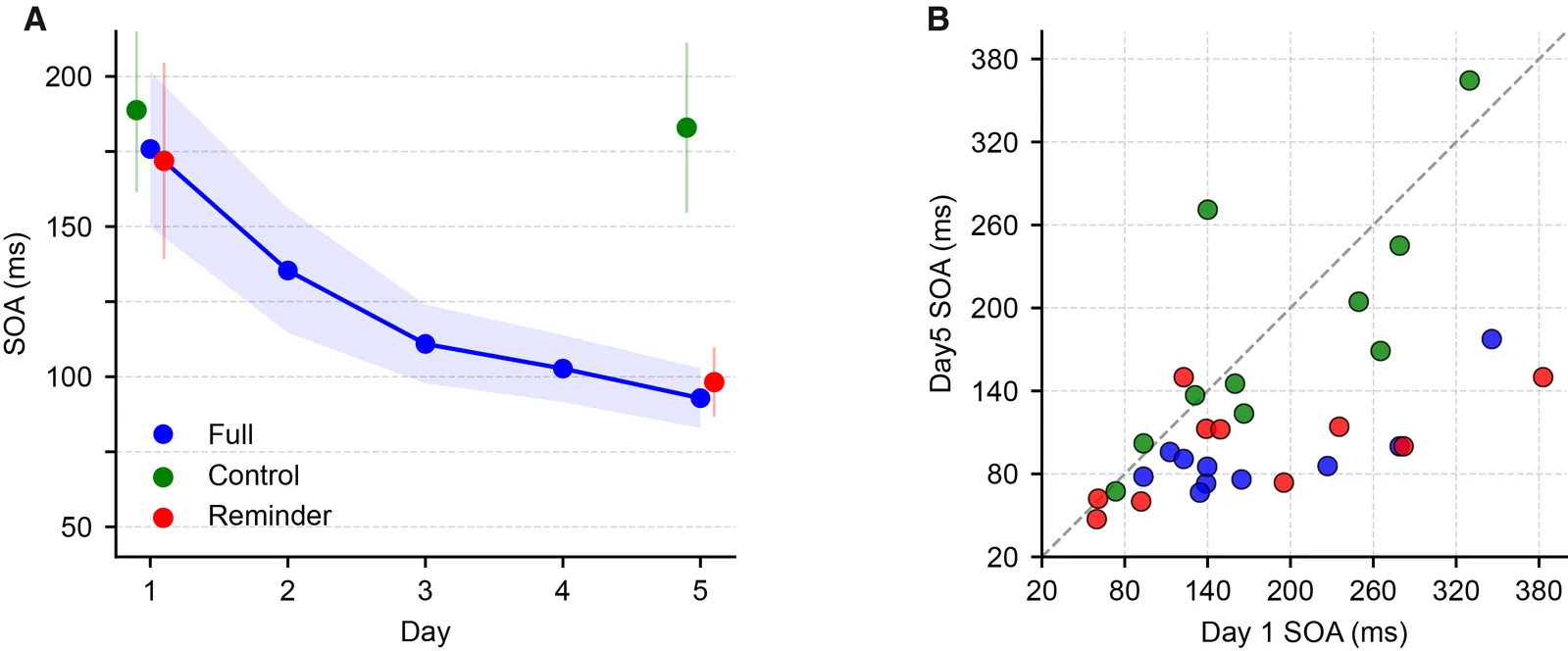
Comparison of learning outcomes between full training (in blue), reminder (in red), and control (in green) conditions in the dual-task TDT (A) Learning curves for the three conditions in the dual-task TDT. Data are represented as mean ±1 SEM. Day 1 thresholds are plotted with a small horizontal offset to display the SEM. (B) Individual participants’ thresholds (81.6% correct) from day 5 plotted as a function of their day 1 thresholds. Data are color-coded as in (A). Dashed diagonal line corresponds to absence of learning; points falling below the diagonal indicate improved performance.

Reminder performance was not significantly correlated with overall learning (r = −0.415, *p* = 0.266), providing no basis for subdividing the reminder condition by reminder strength.^21^ Moreover, participants in the full training condition improved significantly from day 2 to day 5 (t(9) = 3.328, *p* = 0.009), arguing against a test-retest account of the observed improvement.

A Bayesian analysis confirmed that the reminder and full training conditions performed similarly (Bayes factor [*BF*_10_] = 0.409), while the control condition differed from both the reminder (*BF*_10_ = 1.579) and the full training (*BF*_10_ = 5.036) conditions. This strengthened the conclusions from the LMM analysis that the full training and reminder conditions showed learning, whereas the control condition did not.

Finally, we assessed position generalization at an untrained location on day 5 (see STAR Methods). Transfer thresholds were significantly higher than trained-location thresholds in the full training condition (t(8) = -3.95, *p* = 0.004, d = −1.32) but not in the reminder (t(7) = -2.19, *p* = 0.065, d = −0.77) or control condition (t(6) = 0.07, *p* = 0.948, d = 0.03), suggesting greater position specificity following full training.

### Single-task TDT with identical stimuli

Ten participants performed the TDT without the T/L fixation task using the same stimuli as the dual-task TDT. In the absence of a central fixation task, fixation was enforced by eye tracking, whereby any trials in which the gaze shifted beyond 1.5° of the fixation point were aborted and replayed later in the session. On the first day, their mean threshold was 62 ± 15.5 ms (median 53.6), which is substantially lower than the 180 ± 16.8 ms observed on day 1 of the dual-task TDT in the present study and previously reported values in other studies on day 1 (Figure 4; for the parameters of all previous studies see Table S3). The large reduction of SOA thresholds merely by omitting the requirement to perform the central fixation task alongside the peripheral TDT demonstrated that the challenge in the dual-task TDT was mainly attentional. The thresholds in the single-task TDT on day 1 were so low that they left little room for further improvement with training. We therefore abandoned the planned further training in this experiment and instead made the stimulus more challenging to increase the SOA thresholds on day 1 and to enable a sufficient learning amplitude.

**Figure 4:**
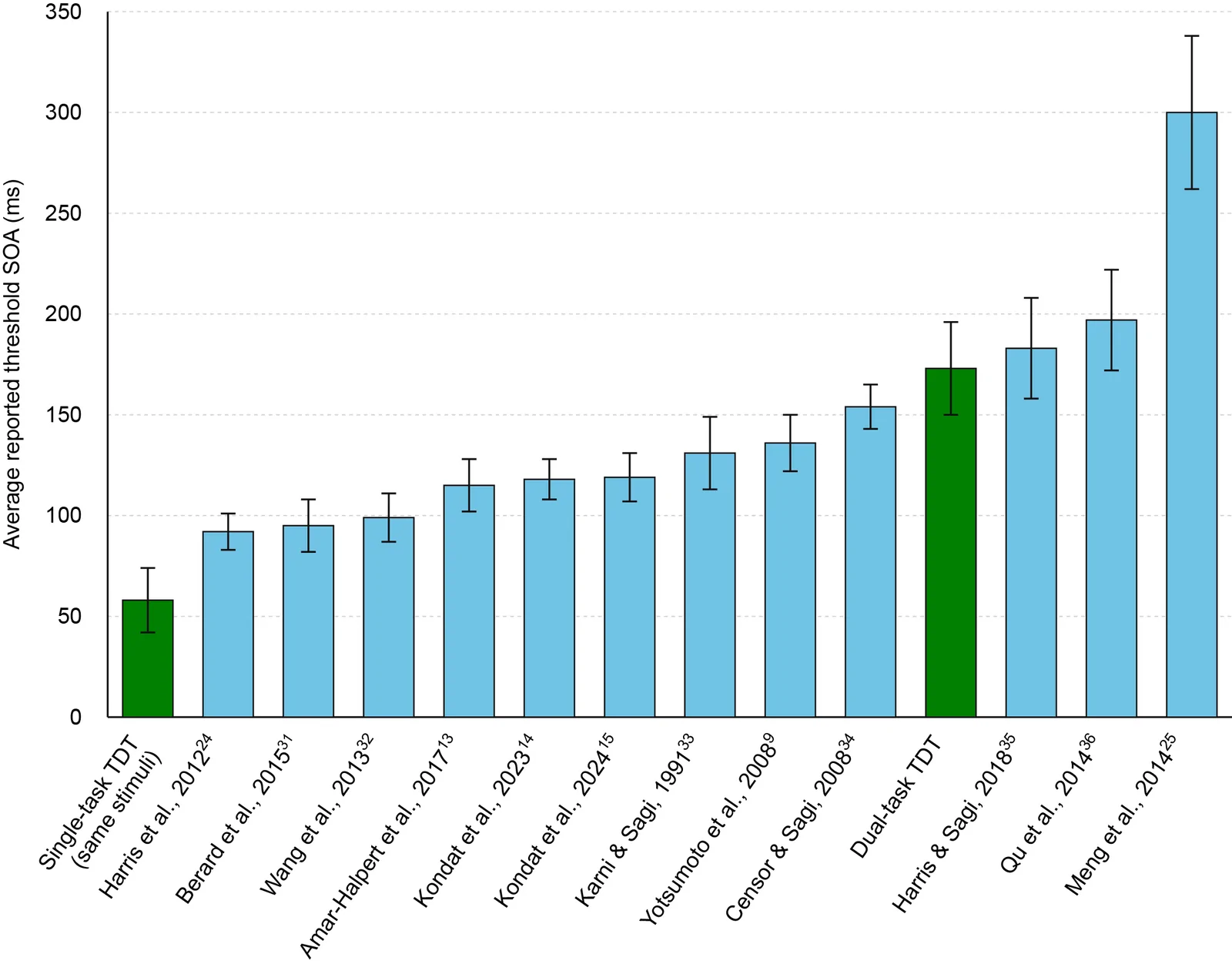
Comparison of day 1 thresholds in the dual-task TDT Comparison of our data to SOA thresholds in dual-task versions of the TDT in existing literature. Light blue bars show SOA thresholds in the standard dual-task version of the TDT in a set of representative studies. Green bars show data from the present study obtained in the dual-task TDT (green bar toward the right) and in the single-task TDT with the same stimulus as used in the dual-task TDT (leftmost bar). The reported thresholds are means ±1 SEM. For the parameters of each study, see Table S3. Superscript numbers following the publication year indicate the corresponding reference number in the present paper’s reference list.

### Single-task TDT with modified stimuli

To increase difficulty, we modified the texture stimulus by increasing the spacing between elements (i.e., making the texture coarser) while maintaining the dimensions of the texture elements. This manipulation is known to increase the difficulty of figure-ground texture segregation and hence the difficulty of the TDT task.^31^^,^^32^ Additionally, the figure was reduced to 2 elements (instead of 3) to further increase difficulty.

Furthermore, after obtaining only 7 complete datasets from a large sample of candidate participants, we slightly adjusted the SOA range for subsequent participants to reduce the number of participants failing to reach the performance level required to continue training after day 1 (see STAR Methods for details). At the same time, pre-training was omitted (see STAR Methods for details) because, in the dual-task TDT, it showed no correlation with day 1 performance (r = −0.217, *p* = 0.373) and thus had no value for early screening of participants who could not perform the task adequately. We retained the familiarization phase. Because 7 participants completed the single-task TDT using the original SOA range and pre-training procedure, whereas the remaining 23 participants completed the modified procedure, we could assess whether these procedural changes affected performance and learning. Learning from day 1 to day 5 did not differ between the two groups (day × group: [*β* = -21.16, SE = 19.225, *p* = 0.271]; *BF*_10_ = 0.26)*.* Moreover, participants tested with and without pre-training showed similar learning trajectories across blocks during day 1 (Figure S1A).

Using the single-task TDT with the modified stimulus, 30 participants completed the experiment (*N* = 10 per condition). Performance on day 1 showed no threshold differences between the three conditions (F(2,54) = 0.018, *p* = 0.98), and learning in the three conditions appeared similar (Figure 5A). Accordingly, the LMM showed no significant time × condition interaction for full training versus control (*β* = −13.061, SE = 20.561, *p* = 0.525), reminder versus control (*β* = −8.169, SE = 20.561, *p* = 0.691), or between full training and reminder (*β* = 4.892, SE = 20.561, *p* = 0.806) (Figure 5A, for single-subject day 1 versus day 5 thresholds see Figure 5B). After removing the interaction terms, the effect of time was significant (*β* = −43.060, SE = 8.161, *p <* 0*.*001). These results indicate that all three conditions showed a similar performance increase (for all LMM effects see Tables S4 and S5). Bayesian analysis confirmed that participants in the three conditions performed similarly (reminder vs. full training (*BF*_10_ = 0.404), control vs. reminder (*BF*_10_ = 0.417), and control vs. full training (*BF*_10_ = 0.505).

**Figure 5:**
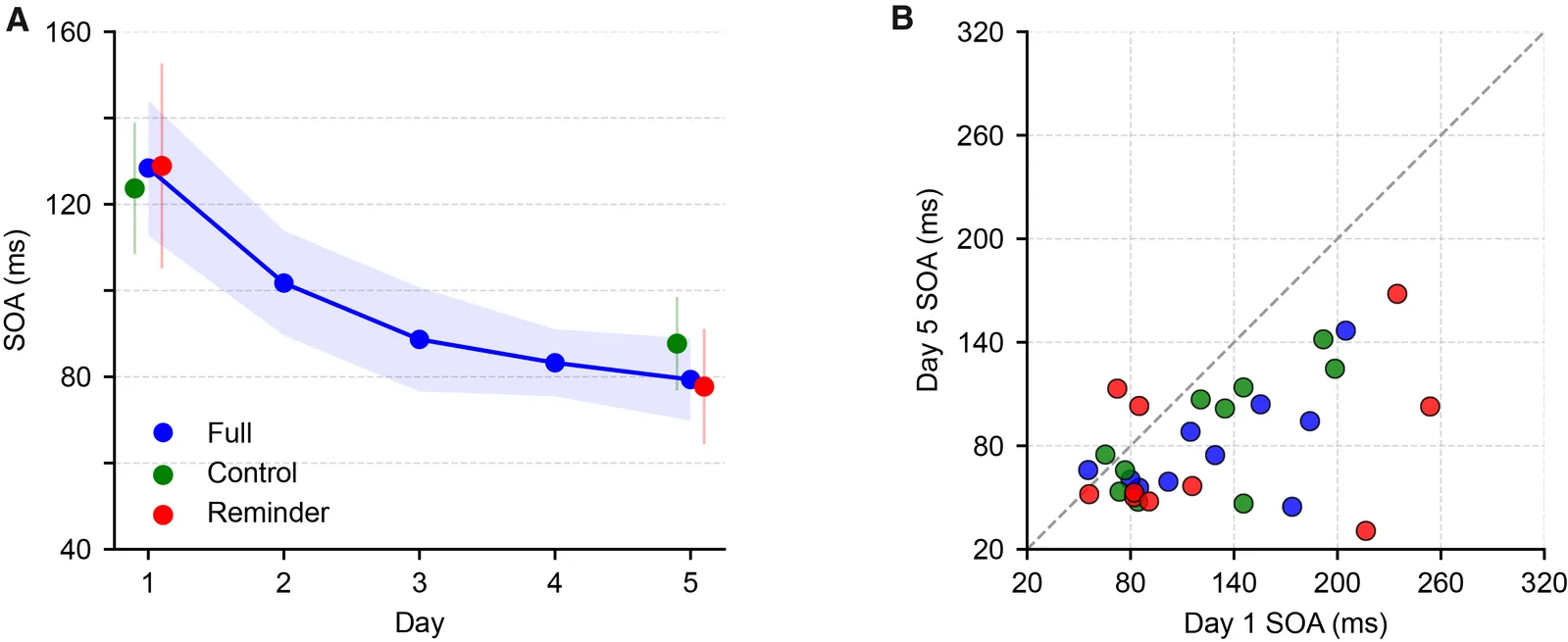
Comparison of learning outcomes between full training (in blue), reminder (in red), and control (in green) conditions in the single-task TDT (A) Learning curves for the three conditions in the single-task TDT. All conventions are the same as in Figure 1. (B) Individual participants' thresholds (81.6%) correct from day 5 plotted as a function of their day 1 thresholds. All conventions are the same as in Figure 1.

Reminder performance was not significantly correlated with overall learning (r = −0.238, *p* = 0.507). Participants in the full training condition improved significantly from day 2 to day 5 (t(9) = 4.36, *p* = 0.002), arguing against a test-retest account of the observed improvement. Position generalization was assessed in a subset of participants on day 5 (see STAR Methods). Across conditions, transfer thresholds were significantly higher than thresholds at the trained location (t(8) = -2.32, *p* = 0.049, d = −0.77), indicating position specificity. The small number of participants with transfer data precluded comparisons between conditions.

To exclude the possibility that the improvement observed on day 5 in the control condition reflected rapid within-session learning, rather than learning occurring between the end of day 1 and the beginning of day 5, we conducted an additional block-wise analysis (Figure S1B). This analysis showed no evidence of systematic within-session improvement on either day. Moreover, a comparison of performance at the end of day 1 with performance at the start of day 5 revealed a significant improvement (t(9) = -3.067, *p* = 0.01343), indicating that the observed gains reflected between-session learning rather than within-session learning.

After the completion of the single-task TDT, we found that—despite stimulus adjustments—there was a remaining trend on day 1 of the control condition toward significantly lower SOA thresholds in the single-task compared to dual-task TDT (t(18) = -2.082, *p* = 0.052). Notwithstanding this trend, there was no significant difference in the amount of learning in the full training condition between the two tasks (t(18) = -0.498, *p* = 0.625, *BF*_10_ = 0.434). This is relevant for the interpretation of differences in results in the other conditions between the two tasks (see discussion).

### Evaluation of gaze bias

We evaluated whether fixation strategies contributed to our learning results. In the dual-task TDT, the T/L task at fixation has been used to enforce fixation, but in principle left participants free to choose another gaze position in an attempt to optimize performance, since previous studies have not recorded eye movements in this task. We had therefore hypothesized that the reported performance increases with training might have been related to participants positioning their gaze closer to the anticipated position of the peripheral target. Our eye movement recordings do not support this idea. Overall, participants fixated close to the fixation spot, with 90% of all trials falling within a 1.2° radius from fixation on day 1 (Figure 6A, left), and within a radius of 1.15° on day 5 (Figure 6A, right). Moreover, there was no significant change in the average distance between the gaze point and the center of the screen from day 1 to day 5 (t(27) = -0.038, *p* = 0.9699) or from the gaze point to the target from day 1 to day 5 (t(27) = 0.0101, *p* = 0.992). Furthermore, we found no consistency in the direction of shift in mean gaze position between days 1 and 5 (z = 1.87, *p* = 0.154) (Figure 6B). This suggests that participants neither fixated on the peripheral target nor adopted a gaze-shifting strategy to improve performance in the dual-task TDT.

**Figure 6:**
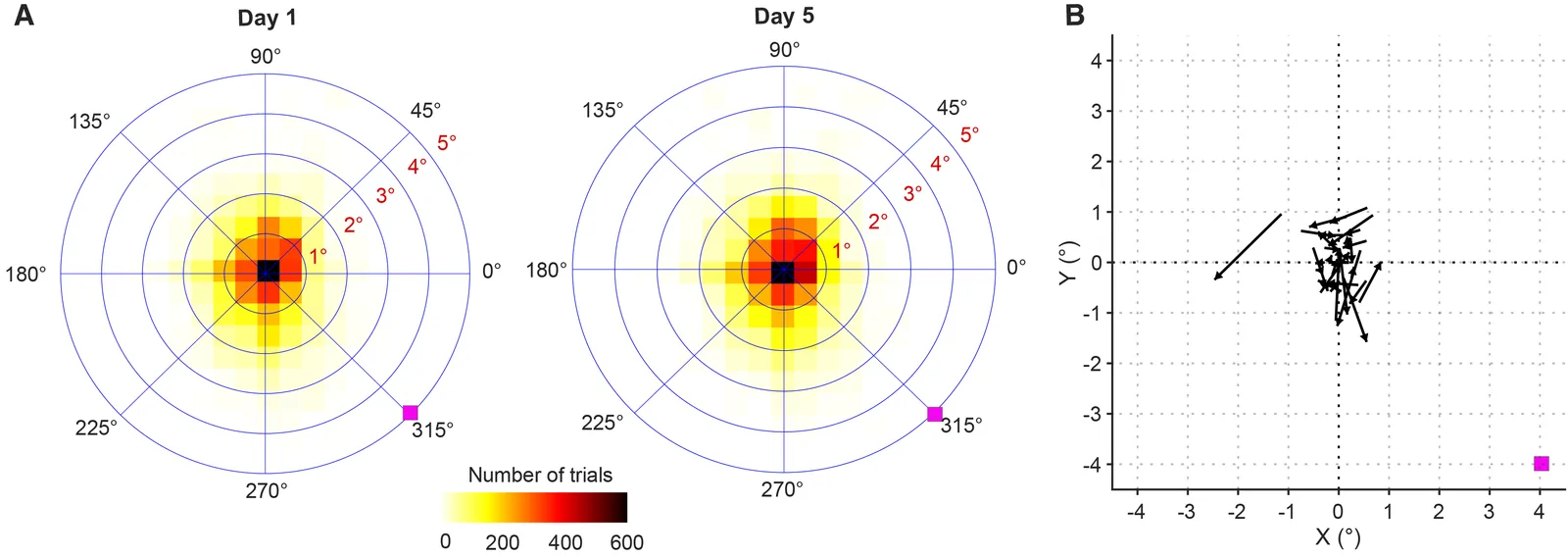
Fixation accuracy in the dual-task TDT (A) Fixation accuracy on day 1 (left) and on day 5 (right). (B) Average X and Y positions per participant on day 1 (start of the arrow) and day 5 (arrowhead). Pink box denotes target position.

In the single-task TDT, where the T/L task was omitted, fixation was continuously enforced with an eye tracker. Trials in which gaze moved outside a circular window of 1.5° around the fixation target were aborted (and replayed later in the experiment). It is possible, however, that the enforcement of fixation with the eye-tracker was less effective than the enforcement of fixation with the behavioral task. Because we did not record the eye movement data during the single-task TDT, we cannot fully exclude a small fixational bias in that experiment. However, moving fixation closer to the edge of the fixation window can be expected to lead to more fixation errors. Instead, average session duration was comparable between the single-task and dual-task TDT (20.0 vs. 22.6 min, respectively, excluding pre-training). Together with other aspects of our data (see discussion), this makes it unlikely that differences in gaze positioning substantially influenced the pattern of learning observed in the single-task TDT.

## Discussion

Amar-Halpert et al.^13^ demonstrated that following an initial training session in a TDT, performance enhancements from full training could be matched by a regimen in which training sessions were replaced by just a few reminder trials (reminder condition). The reminder trials were crucial because omitting them (control condition) prevented learning. In the TDT task, participants simultaneously discriminated both a central target (T vs. L) and a peripheral target (horizontal versus vertical), which limited attention to the target (dual-task TDT). Here, we asked whether reminder effectiveness depends on limited attention to the target and report three main findings. First, we provided a direct replication of Amar-Halpert et al.’s^13^ finding of reminder benefits in the dual-task TDT and furthermore showed that the effect was not explained by strategic eye movements. Second, in a single-task version of the TDT with the same stimuli (but without the fixation task), we observed very low thresholds already on day 1, indicating near-ceiling performance that left little room for further improvement. To enable learning in the single-task TDT, we enhanced the difficulty of the stimulus configuration by increasing the spacing between elements and reducing the number of target elements. Finally, using this modified stimulus in the single-task TDT, we trained three groups in the same three conditions as used in the dual-task TDT and discovered that unconstrained attention to the target during the first training session triggered learning that proceeded for several days without reminders or any further training. We interpret our data as support for the idea that attentional context (defined here as the task configuration that determines how many spatial locations must be selected and attended) is a key factor shaping whether an initial training session triggers a learning process that can unfold over several days in the absence of any further practice or a learning process benefiting from full standard training or reminders.^13^^,^^14^^,^^15^^,^^20^^,^^21^ Strikingly, in neither setting did full standard training offer a performance benefit over reminder-only training.

In the dual-task TDT, we replicated Amar-Halpert et al.^13^’s core finding that reminders can replace large amounts of training trials. Unlike in their study, we equated the time difference between the first and last session across all three experimental conditions, which renders the evidence for reminder effectiveness in our study arguably even stronger and adds to the evidence questioning the relevance of daily training. Additionally, our eye-tracking data ruled out anticipatory gaze shifts toward the target as a source of improvement. Amar-Halpert et al.^13^ attributed reminder benefits to reconsolidation—a process by which retrieved memories become labile and need to be reconsolidated.^19^^,^^33^ Reconsolidation can be disrupted by behavioral interference,^34^ stress,^35^^,^^36^ the inhibition of protein synthesis,^33^ or stimulation.^37^^,^^38^^,^^39^ Yet, the lability of the memory trace during reconsolidation may also allow it to become strengthened through attention, consistent with related evidence that attention strengthens consolidation.^40^

In the single-task TDT, we suggest that removing the central task increased the availability of attentional resources for the peripheral target. In line with this, the single-task TDT with the original stimulus resulted in very low SOA thresholds already on day 1, comparable to those observed in the dual-task TDT only after several days of training (Figure 4). Hence, our data and prior literature^26^^,^^27^ suggest that more processing resources were available for the peripheral target in the single-task than in the dual-task TDT. The limited availability of attention for target discrimination likely applied also to other studies that used the dual-task TDT.^3^^,^^13^^,^^14^^,^^22^^,^^31^ The pattern of data shown in our study indicates that performance in the dual-task condition was primarily limited by attentional factors rather than by sensory constraints.

The near-ceiling level performance in the single-task TDT following removal of the requirement to perform the central fixation task necessitated a modification of the stimulus to enable sufficient learning across days. To that aim, we increased task difficulty in the single-task TDT by sparsening the texture. This manipulation reduces crowding,^41^ and concomitantly weakens the pooling mechanisms that contribute to texture segregation. In the texture stimuli we used, these pooling mechanisms involve iso-orientation inhibition among uniformly oriented background elements, which suppresses background activity and causes a release of inhibition in the figure region, underlying the figure’s salience. Increasing spacing reduces this mutual inhibition, which decreases target detectability.^42^^,^^43^^,^^44^^,^^45^ To further enhance difficulty in the single-task TDT, we also reduced the target to two texture elements to decrease its detectability and its discriminability. Using this modified stimulus, we trained participants in the same three conditions as in the dual-task TDT (full training, reminder, and control).

In the single-task TDT with the modified stimulus, the comparable learning in control and full training conditions suggests that the performance improvement following a single training session may last substantially longer than previously assumed and is affected by neither reminder trials nor extensive daily training. Most skill learning studies employ daily training schedules,^3^^,^^4^^,^^5^^,^^6^^,^^7^^,^^8^^,^^9^^,^^10^^,^^11^^,^^12^^,^^13^ thereby implicitly assuming that offline learning processes do not extend beyond a single day. Consequently, potential offline learning processes occurring during periods without task exposure longer than a day are often not examined directly. Similarly, interference studies^46^^,^^47^ have typically estimated consolidation durations to be in the order of a few hours, and sleep studies^48^^,^^49^ have focused on overnight consolidation. Stickgold, James, and Hobson,^23^ however, demonstrated in a dual-task TDT that SOA thresholds decreased by approximately 20 ms when participants were retested 3 to 4 days after an initial training session, with no intervening sessions, supporting a protracted consolidation process. Our control data in the single-task TDT showed similar SOA reductions of about 36 ms on day 5. Notably, an analysis that compared across-session learning with within-session learning confirmed that the reduction of the session averages from day 1 to day 5 reflected genuine latent consolidation. Our results therefore suggest that behaviorally relevant improvements unfold over multiple days. Importantly, however, because control participants were only retested on day 5, our experimental design does not allow us to distinguish among the possibilities that the improvements reflected a gradual multi-day consolidation process or a fast consolidation process occurring primarily during the first night after training. To more definitively establish whether consolidation unfolds progressively across multiple days, future studies should include additional control groups, each tested at different intermediate time points.

Because of the hypothesized link between weak memory traces and limited target-directed attention, we had predicted that the beneficial effect of reminders would be restricted to the dual-task TDT. At face value, our data confirm this. Nevertheless, the unexpectedly strong learning in the control condition of the single-task TDT meant that there was also no room for the reminders to outperform it. Therefore, it can be argued that we did not fully demonstrate that reminders do not offer added benefits in a single-task TDT permitting full attention to the target. Nevertheless, we believe that our findings and those of others^13^^,^^14^^,^^15^ are consistent with the view that attentional context and task demands determine under which conditions reminders can be effective. If this perspective is correct, then it may explain a recent report by Zhu and Zhang.^50^ The authors observed comparable learning in a dual-task TDT across full training, control, and reminder conditions, mirroring our findings in the single-task TDT. Therefore, a potential explanation for Zhu and Zhang’s^50^ findings is that their participants had more attention available for the target than in preceding dual-task TDT studies. Differences in the size of the T/L stimuli at fixation, or differences among participant pools in pre-existing perceptual skills (e.g., due to gaming experience) and motivation (e.g., due to differences in instruction or in the perceived value of the reward) could all have modulated attentional allocation to the texture target in such a manner as to mimic our single-task TDT results. A related finding was reported by Chen and Op de Beeck.^51^ Using a complex-object perceptual learning task, they compared full training and reminder learning and found that although reminders produced some improvement, these gains were substantially smaller than those elicited by repeated, intensive training. Their results indicate that reminder-induced learning effects may not generalize to tasks that involve more extensive perceptual encoding or attentional engagement, aligning with our interpretation that the attentional resources available for the target critically modulate whether reminder benefits can emerge.

In summary, in a 5-day visual perceptual learning task with restricted processing resources available for the peripheral target, reminder trials following an initial training session successfully reduced daily practice to just a few trials per day without compromising learning amplitude. In contrast, in a similar task context with reduced overall demands on attention and cognitive control, performance improvements emerged that were equivalent to those yielded by several days of intensive practice, even in the complete absence of additional practice. Together, these findings invite a reconsideration of the assumption that repeated daily training sessions containing large numbers of trials are inherently necessary to achieve perceptual learning. Rather, our results suggest that the effectiveness of reminders—and the extent to which additional practice is required—depends on the broader task context, including attentional and cognitive demands, and stimulus conditions. Future research should further clarify the temporal dynamics of these improvements and determine whether similar effects extend to later, asymptotic stages of learning.

### Limitations of the study

Beyond the lack of control conditions required to assess the precise time course of consolidation in the single-task TDT, our study has several limitations. First, we interpret the different results between the dual-task and single-task experiments primarily in terms of the difference in attentional context. When the peripheral target discrimination is performed concurrently with the letter discrimination at fixation, much less attention can be allocated to the peripheral target discrimination than when the fixation task is omitted.^26^ We suggest that how much attention can be allocated to the peripheral target discrimination is the primary factor underlying the different result patterns in the dual and single-task TDTs. However, the manipulation of attentional context likely affected additional, related processes that also could have influenced task performance. In particular, performing two tasks instead of one may increase executive (cognitive control) demands associated with selecting and processing information at multiple spatial locations and may also place modest demands on short-term maintenance processes. Furthermore, the present behavioral experiment could not resolve the manner in which attention was deployed across locations—whether through parallel allocation or rapid sequential shifts^26^—and how potential changes in attentional allocation might have played a role in the learning behavior observed in our experiments.

In addition, although we did not discover gaze biases in the dual-task TDT, they might have been present in the single-task TDT, where we restricted eye movements within a small window surrounding the fixation spot but did not record the eye movements. We consider the possibility of a gaze bias unlikely because significant, systematic biases would have led to a substantial increase in fixation errors, which was not supported by our data. In addition, it could be argued that microsaccades within the fixation window in the single-task TDT might have had specific properties that could have explained the difference in learning results compared to the dual-task TDT. However, we suggest that, should there have been a difference in microsaccade patterns in single-task compared to dual-task TDT, its most parsimonious interpretation would have been that it reflects differences in attentional allocation between the two TDT versions—in line with our experimental manipulation. Moreover, even if participants in the single-task TDT used microsaccades to slightly shift gaze toward the target upon stimulus appearance, previous studies using similar texture discrimination paradigms indicate that performance remains largely stable across an eccentricity range (approximately 4°–9°) that includes the target eccentricity used in our experiments (5.72°). This makes it unlikely that small retinal displacements produced by microsaccades or gaze biases within the 1.5° fixation window would have substantially affected discrimination performance.^52^^,^^53^^,^^54^

Finally, although the stimulus manipulation introduced in the single-task TDT was intended to compensate for the substantial performance benefit associated with removing the fixation task, we cannot completely exclude that the trend toward a remaining performance advantage in the single-task compared to the dual-task contributed to the observed learning effects. Specifically, the lower thresholds observed in the single-task TDT may have facilitated stronger initial encoding and subsequent offline consolidation and could have contributed to the different patterns of results in the single-task and dual-task TDT. A finding softening this point is that the amount of learning in the full training condition of the single-task did not differ statistically from that in the dual-task. This argues against the idea that strong encoding differences in the first training session between single-task and dual-task TDT explain the different patterns of results in the three conditions of these two experiments. To the extent encoding differences did or did not play a role, the present experiments do not allow us to disentangle them from the stimulus-, task-, and attention-related factors manipulated in our experiments, and future work will be required to determine their relative contributions to the observed learning effects.

## Resource availability

### Lead contact

Requests for further information and resources should be directed to and will be fulfilled by the lead contact, Ylka Kolken (ylka.kolken@maastrichtuniversity.nl).

### Materials availability

This study did not generate new, unique reagents.

### Data and code availability

• Anonymized data have been deposited in the Open Science Framework (OSF) and are publicly available as of the date of publication. Accession numbers are listed in the key resources table.
• All original code has been deposited in the Open Science Framework (OSF) and is publicly available as of the date of publication. Accession numbers are listed in the key resources table.
• Any additional information required to reanalyze the data reported in this paper is available from the lead contact upon request.

## Acknowledgments

The authors would like to thank the students of group 47 and group 49 of academic year 2023–2024. This work is supported by funds from the 10.13039/501100003246NWO Open Competition grant (406.21.GO.044) to P.D.W.

## Author contributions

N.C. developed the original dual-task TDT code, to which M.J.R. incorporated the eye-tracking components. The concept for the single-task TDT was developed by Y.K. and P.D.W., with additional programming support from M.J.R. Y.K. collected the data. Statistical consultation was provided by P.N. and F.D.M. The manuscript was written by Y.K. and P.D.W., and all authors reviewed and edited the final version.

## Declaration of interests

The authors declare no competing interests.

## STAR★Methods

### Key resources table

**Table:** 

REAGENT or RESOURCE	SOURCE	IDENTIFIER
**Software and algorithms**		

MATLAB R2024a	N/A	https://www.mathworks.com
Python	N/A	https://www.python.org

**Deposited data**		

Generated data and code	OSF	https://osf.io/gqcbn/

### Experimental model and study participant details

One hundred three volunteers (mean age 22.5 ± 1.53, 64 female) naive to the purpose of the experiment participated in the different experiments (for the distribution of participants among experiments, see Table S1). Sex was not included as a factor in the statistical analyses, precluding conclusions regarding sex-related differences. Each experiment included three conditions (Figure 2): a *full training* condition, in which participants trained for five consecutive days; a *reminder* condition, in which participants trained on day 1 and day 5 and received five near-threshold reminder trials on days 2–4; and a *control* condition in which no training occurred on days 2–4. For all participants, the interval between the first (day 1) and the final session (day 5) was exactly 4 days. Participants were not informed about the experimental design or aims. Participants were randomly assigned to groups using a computer-generated randomization procedure, with group assignment disclosed only after the first training session. This ensured that all participants began the experiment with equal motivation and without bias from prior knowledge of their training schedule.

### Method details

#### Stimuli and tasks

We adopted the setup of Amar-Halpert et al.^13^ with the only difference being a longer stimulus duration (17 ms instead of 10 ms) due to the lower refresh rate of our screens. Participants discriminated the orientation of a texture-defined target stimulus presented in the lower-right visual quadrant (Figure 1A). The target stimulus consisted of three diagonal bars (tilted 135°), presented in the lower right quadrant of the visual field at 5.72° eccentricity (x = 4.042°, y = −4.042°, Figure 1B). During transfer testing on Day 5, the texture-defined target appeared in the upper-left quadrant at the same eccentricity (x = −4.042°, y = 4.041°). The background array comprised 19 × 19 horizontal bar elements (0.57 ° × 0.04 °), spaced 0.86° apart with 0.04° jitter. The stimulus subtended 15.4 ° × 15.1 ° (24-inch Iiyama Prolite PC monitor, 60Hz refresh rate, 1920 × 1080-pixel resolution, mean texture luminance 84 cd/m2), viewed from 65 cm distance. All target durations, SOAs, and the mask duration were verified with a photodiode.

In the dual-task TDT, fixation was enforced by a forced-choice letter discrimination task (T or L at the center of the display) with auditory feedback, in line with previous studies using this paradigm.^3^^,^^9^^,^^14^ Eye movements were recorded using a Viewpoint Eye Tracker (Arrington Research, Scottsdale, AZ, USA). At the beginning of each trial, participants fixated their gaze on the center of the screen and pressed the space bar to start the trial (Figure 1A). After the appearance of a fixation cross, a target frame (17 ms) was followed (after a given SOA) by a patterned mask consisting of randomly oriented V-shaped elements and a central composite pattern of superimposed T and L shapes (102 ms). After the stimulus presentation, participants reported the perceived letter and target orientation with separate keyboard presses; first they reported the T or L fixation target using ‘T’ or ‘L’ keys, which was followed by auditory feedback in the case of incorrect response. Second, they reported the target orientation, using ‘T’ for ‘horizontal’ and ‘L’ for ‘vertical’, which was not followed by feedback. There was no response time limit.

Prior to the experiment, participants underwent a pre-training phase followed by a familiarization phase. In the pre-training phase, participants were given a maximum of ten blocks, each containing ten trials, with the aim of reaching a level of 90% correct responses in a block at the highest SOA (323 ms) for both the fixation and the texture discrimination tasks. If the participant did not reach this level of accuracy within 10 blocks, the participant was dismissed. The familiarization phase consisted of a block of trials, presenting one trial per SOA. The intervals between the target and the mask onset ranged from 17 ms to 323 ms in increments of 17 ms and were randomized across all trials [17 51 68 85 102 119 153 170 187 204 221 255 289 and 323]. Participants who did not reach the 81.6% correct threshold after pre-training and familiarization on day 1 were excluded from further participation. These criteria (not passing the pre-training or not reaching an 81.6% threshold on day 1) led to the exclusion of 15 participants. Of the 30 included participants, three in *full training* were trained with 13 SOA levels [51 68 85 102 119 153 170 187 204 221 340 391 and 425] instead of 14 (S3 on all days, S12 only on day 1, S5 on day 3) due to a video card error, and one participant in *full training* performed only 7 blocks on day 1 instead of 9 due to time pressure (S14).

In the single-task TDT, we used the same setup as in the dual-task TDT, except that the T/L task was omitted. In the absence of a central fixation task, fixation was enforced using an eyetracker (Arrington Research, Scottsdale, AZ, USA). If the participants’ gaze deviated *>* 1.5° away from the center of the screen at any point during a 1-s interval prior to stimulus presentation, or during stimulus presentation, the trial was aborted and replaced by another trial. An automatic recalibration of the eye tracker would begin if participants broke fixation on 5 consecutive trials.

Ten participants performed the single-task TDT with the same stimuli as used in the dual-task TDT in a single session. The single-task TDT was also performed with a modified stimulus, which consisted of a 13 × 13 element texture array (line elements of 0.57 × 0.04°, spaced 1.2° apart with 0.05° jitter; see Figure 1C), instead of 19 × 19 array, and 2-element targets instead of 3-element targets. These changes were motivated by preliminary data, which showed near-maximum performance could be reached in the first session with the original stimulus. With the modified stimulus, we trained participants in the same three conditions (*full training*, *reminder*, and *control*) as in our dual-task TDT.

After testing 15 participants in the single-task TDT with the modified stimulus on day 1, and excluding 8, two additional changes were made relative to the dual-task TDT to reduce exclusions on day 1. First, to reduce the training time on day 1, the pre-training task was omitted, as it showed no correlation with day 1 performance in the dual-task TDT (r = −0.217, *p* = 0.373) and added no value beyond the familiarization task. Second, the SOA range was adjusted (51–425 ms instead of 17–323 m). Hence, of the first 15 participants we tested with pretraining and the original SOA range on day 1, 8 failed to reach the 81.6% threshold and were excluded, which left 7 participants who had the original SOA range and who completed pre-training for inclusion in the final analyses. With the modified SOA range [51–425 ms, 13 levels] and without pretraining, only 2 of 25 were excluded.

#### Sample size

To determine the required sample size for the dual-task TDT, we based our estimation on the *a priori* difference between the *full training* and *control* conditions, as a sufficiently robust difference between *control* and *full training* conditions is a prerequisite for a meaningful comparison of the *reminder* condition with these two conditions. In the study by Amar-Halpert et al., 2017,^13^ the mean relative improvement in perceptual thresholds from day 1 to day 5 was 26.6% for the *full training* condition (*N* = 12) and 6.3% for the *control* condition (*N* = 7) (assuming similar day 1 thresholds, which were not reported). Based on the reported group-level variability, we estimated a pooled standard deviation of 17%, yielding a corresponding Cohen’s d ≈ 1.17 for the group difference. An *a priori* power analysis conducted in G∗Power (Version 3.1)^55^ indicated that detecting this effect size with 80% power (1-*β*) at *α* = 0.05 would require 10 participants per condition, which we therefore adopted in our design. For the single-task TDT, we used the same effect-size estimate and applied the same rationale and likewise planned for 10 participants per condition.

### Quantification and statistical analysis

#### Threshold calculation

The psychometric function was modeled using the Weibull sigmoid, scaled to account for guessing and lapses, following the form used in the psignifit4 toolbox:^56^

ψ(x;m,w,λ,γ)=γ+(1−λ−γ)·S(x;m,w)

where threshold *m* is the SOA level at which 81.6% of responses are correct; *w* represents the width; *λ* represents the lapse rate (free); and *γ* represents the guess rate, fixed at 0.5.

#### Statistics

Frequentist analyses were performed in Python 3.10,^57^ Bayes factor calculations in JASP,^58^ and eye-tracking analyses in MATLAB.^59^ To check for baseline differences, we first conducted a one-way ANOVA on the day 1 thresholds to test for condition differences. To evaluate both within-condition learning and between-condition differences in learning across the *full training*, *reminder*, and *control* conditions, we fit a linear mixed-effects model (LMM). This model included fixed effects of Time (day 1 vs. day 5), Condition, and their interaction (Time × Condition), allowing us to assess overall changes in performance, condition differences in baseline performance, and whether learning (i.e., performance change over time) varied by condition. Random intercepts and slopes for Time were included for each subject to account for individual differences in initial performance and learning rate. The model was specified as follows:

$$score_{\left\{ij\right\}}=\beta_{0}+\beta_{1}\times Time_{\left\{ij\right\}}+\beta_{2}\times Condition2+\beta_{3}\times Condition3+\beta_{4}\times \left(Time_{\left\{ij\right\}}\times Condition2\right)+\beta_{5}\times \left(Time_{\left\{ij\right\}}\times Condition3\right)+u_{\left\{0i\right\}}+u_{\left\{1i\right\}\;}\times Time_{\left\{ij\right\}}+\varepsilon_{\left\{ij\right\}}$$

where score_*ij*_ and time_*ij*_ represent respectively the performance score (in ms) and the measurement time for the *j*th measurement of the *i*th participant. *Condition* stands for the three conditions (*control* is condition 1 (baseline), *full training* is condition 2, and *reminder* is condition 3). The fixed effects are represented by *β* coefficients: *β*_0_ is the overall intercept (i.e., the grand mean performance), *β*_1_ captures the average effect of Time (change from day 1 to day 5), *β*_2_ and *β*_3_ represent the effect of Condition, and *β*_4_ and *β*_5_ capture the interaction between Time and Condition, which reflects differences in learning across conditions. The terms *u*_0*i*_ and *u*_1*i*_ are subject-specific random effects: *u*_0*i*_ is a random intercept accounting for individual differences in baseline performance, and *u*_1*i*_ is a random slope allowing for individual variability in learning rate (i.e., change over time). The residual error term *ε*_*ij*_ is a within-individual measurement error that captures unexplained variance at each observation. Together, these components allow the model to flexibly account for both fixed effects of experimental factors and random variation across individuals. Model assumptions were evaluated by inspecting residuals against fitted values to ensure homoscedasticity and normally distributed errors.

In addition to the frequentist mixed-effects model, we conducted a Bayesian ANOVA on normalized day 5 scores (calculated as day 5 performance minus day 1 performance) to assess condition differences in learning. This analysis was performed using JASP (version 0.19) with default priors: a zero-centered Cauchy (0, r = 1/ $\sqrt{2}$ ≈ 0.707) for fixed effects and a Jeffreys prior for variance components which lead to non-informative priors. The Bayesian approach permits quantifying the relative evidence for or against the presence of condition differences by computing Bayes factors BF, offering a complementary interpretation to traditional null hypothesis significance testing. The magnitude of the Bayes Factor reflects the strength of evidence.^60^^,^^61^^,^^62^ We report *BF*_10_, which quantifies the evidence in favor of the alternative hypothesis (H1) relative to the null hypothesis (H0). A *BF*_10_ *>* 1 indicates support for the alternative hypothesis, whereas A *BF*_10_ *<* 1 indicates support for the null hypothesis.

In the dual-task TDT, we quantified changes in gaze behavior over time using the eye-tracking data collected on day 1 and day 5 of the experiment. For each participant in these two sessions, we computed the distance of the average gaze position from the center of the screen to assess potential shifts in overall gaze centering across sessions. Second, we calculated the distance between the average gaze position and the target location, to see if, over time, participants shifted their gaze toward the target. To evaluate whether these measures changed over time, we performed a paired samples t-tests comparing day 1 and day 5 across participants. We used circular statistics to test whether gaze shifts were consistent in direction across participants by submitting day 1–day 5 movement angles to a Rayleigh test of non-uniformity. Fixation behavior was stored only in the dual-task TDT and not in the single-task TDT, which restricted this analysis to the dual-task TDT experiment. Location transfer within each condition was assessed using paired t-tests in the dual-task TDT.

For the single-task TDT, seven participants were trained using the original 14-level SOA range and received a pre-training session, whereas 23 participants were trained using a modified 13-level SOA range without pre-training. This warranted several control analyses. To assess whether these procedural differences affected baseline performance or learning, threshold estimates were analyzed using an LMM with Time (day 1 vs. day 5), Group (14-level SOA range with pre-training vs. 13-level SOA range without pre-training), and their interaction as fixed effects, with random intercepts and slopes for participants. In addition, learning trajectories across blocks on day 1 were compared between groups to assess whether pre-training affected within-session learning. Finally, to distinguish within-session from between-session learning in the *control* condition, threshold estimates were calculated separately for each block on days 1 and 5 and analyzed using an LMM with Day, Block, and their interaction as fixed effects and a random intercept for participant. The Day effect assessed differences in performance between sessions, the Block effect assessed within-session changes in performance, and the Day × Block interaction assessed whether within-session learning differed between days.

## References

[1] James W. (1890).

[2] Stratton G.M. (1896). The new psychological laboratory at Leipzig. Science.

[3] Karni A., Sagi D. (1991). Where practice makes perfect in texture discrimination: evidence for primary visual cortex plasticity. Proc. Natl. Acad. Sci. USA.

[4] Gibson J.J., Gibson E.J. (1955). Perceptual learning: differentiation or enrichment?. Psychol. Rev..

[5] McKee S.P., Westheimer G. (1978). Improvement in vernier acuity with practice. Percept. Psychophys..

[6] Fahle M., Edelman S. (1993). Long-term learning in vernier acuity: effects of stimulus orientation, range and feedback. Vision Res..

[7] Schoups A.A., Vogels R., Orban G.A. (1995). Human perceptual learning in identifying the oblique orientation: retinotopy, orientation specificity and monocularity. J. Physiol..

[8] Seitz A.R., Yamagishi N., Werner B., Goda N., Kawato M., Watanabe T. (2005). Task-specific disruption of perceptual learning. Proc. Natl. Acad. Sci. USA.

[9] Yotsumoto Y., Watanabe T., Sasaki Y. (2008). Different dynamics of performance and brain activation in the time course of perceptual learning. Neuron.

[10] Gilbert C.D., Li W., Piech V. (2009). Perceptual learning and adult cortical plasticity. J. Physiol..

[11] Lu Z.L., Lin Z., Dosher B.A. (2016). Translating perceptual learning from the laboratory to applications. Trends Cogn. Sci..

[12] Watanabe T., Sasaki Y. (2015). Perceptual learning: toward a comprehensive theory. Annu. Rev. Psychol..

[13] Amar-Halpert R., Laor-Maayany R., Nemni S., Rosenblatt J.D., Censor N. (2017). Memory reactivation improves visual perception. Nat. Neurosci..

[14] Kondat T., Aderka M., Censor N. (2023). Modulating temporal dynamics of performance across retinotopic locations enhances the generalization of perceptual learning. iScience.

[15] Kondat T., Tik N., Sharon H., Tavor I., Censor N. (2024). Distinct neural plasticity enhancing visual perception. J. Neurosci..

[16] Walker M.P., Brakefield T., Hobson J.A., Stickgold R. (2003). Dissociable stages of human memory consolidation and reconsolidation. Nature.

[17] Lee J.L.C. (2008). Memory reconsolidation mediates the strengthening of memories by additional learning. Nat. Neurosci..

[18] Dudai Y. (2012). The restless engram: consolidations never end. Annu. Rev. Neurosci..

[19] Nader K. (2015). Reconsolidation and the dynamic nature of memory. Cold Spring Harb. Perspect. Biol..

[20] Schrift G., Dotan D., Censor N. (2022). Brief memory reactivations induce learning in the numeric domain. npj Sci. Learn..

[21] Herszage J., Bönstrup M., Cohen L.G., Censor N. (2023). Reactivation-induced motor skill modulation does not operate at a rapid micro-timescale level. Sci. Rep..

[22] Karni A., Sagi D. (1993). The time course of learning a visual skill. Nature.

[23] Stickgold R., James L., Hobson J.A. (2000). Visual discrimination learning requires sleep after training. Nat. Neurosci..

[24] Harris H., Gliksberg M., Sagi D. (2012). Generalized perceptual learning in the absence of sensory adaptation. Curr. Biol..

[25] Meng X., Lin O., Wang F., Jiang Y., Song Y. (2014). Reading performance is enhanced by visual texture discrimination training in Chinese-speaking children with developmental dyslexia. PLoS One.

[26] Jans B., Peters J.C., De Weerd P. (2010). Visual spatial attention to multiple locations at once: the jury is still out. Psychol. Rev..

[27] Kastner S., De Weerd P., Desimone R., Ungerleider L.G. (1998). Mechanisms of directed attention in the human extrastriate cortex as revealed by functional MRI. Science.

[28] Mukai I., Bahadur K., Kesavabhotla K., Ungerleider L.G. (2011). Exogenous and endogenous attention during perceptual learning differentially affect post-training target thresholds. J. Vis..

[29] Hoffman J.E. (1998). Visual attention and eye movements.

[30] Schroth L.S.M., Fias W., Sahan M.I. (2025). Eye movements follow the dynamic shifts of attention through serial order in verbal working memory. Sci. Rep..

[31] Scialfa C.T., Jenkins L., Hamaluk E., Skaloud P. (2000). Aging and the development of automaticity in conjunction search. J. Gerontol. B Psychol. Sci. Soc. Sci..

[32] Pavlovskaya M., Ring H., Groswasser Z., Keren O., Hochstei S. (2001). Visual search in peripheral vision: learning effects and set-size dependence. Spat. Vis..

[33] Nader K., Schafe G.E., LeDoux J.E. (2000). Fear memories require protein synthesis in the amygdala for reconsolidation after retrieval. Nature.

[34] Schroeder M.N., Fullio C.L., Ballarini F., Moncada D. (2023). Modulation of memory reconsolidation by adjacent novel tasks: timing defines the nature of change. Commun. Biol..

[35] McGaugh J.L. (2004). The amygdala modulates the consolidation of memories of emotionally arousing experiences. Annu. Rev. Neurosci..

[36] Cai W.H., Blundell J., Han J., Greene R.W., Powell C.M. (2006). Pos treactivation glucocorticoids impair recall of established fear memory. J. Neurosci..

[37] Misanin J.R., Miller R.R., Lewis D.J. (1968). Retrograde amnesia produced by electroconvulsive shock after reactivation of a consolidated memory trace. Science.

[38] Robertson E.M. (2012). New insights in human memory interference and consolidation. Curr. Biol..

[39] Shmuel D., Frank S.M., Sharon H., Sasaki Y., Watanabe T., Censor N. (2021). Early visual cortex stimulation modifies well-consolidated perceptual gains. Cereb. Cortex.

[40] Kentros C.G., Agnihotri N.T., Streater S., Hawkins R.D., Kandel E.R. (2004). Increased attention to spatial context increases both place field stability and spatial memory. Neuron.

[41] Bouma H. (1970). Interaction effects in parafoveal letter recognition. Nature.

[42] Nothdurft H.C. (1985). Orientation sensitivity and texture segmentation in patterns with different line orientation. Vision Res..

[43] Knierim J.J., Van Essen D.C. (1992). Visual cortex: cartography, connectivity, and concurrent processing. Curr. Opin. Neurobiol..

[44] Lamme V.A., Rodriguez-Rodriguez V., Spekreijse H. (1999). Separate processing dynamics for texture elements, boundaries and surfaces in primary visual cortex of the macaque monkey. Cereb. Cortex.

[45] Self M.W., van Kerkoerle T., Supèr H., Roelfsema P.R. (2013). Distinct roles of the cortical layers of area V1 in figure–ground segregation. Curr. Biol..

[46] Brashers-Krug T., Shadmehr R., Bizzi E. (1996). Consolidation in human motor memory. Nature.

[47] Shadmehr R., Brashers-Krug T. (1997). Functional stages in the formation of human long-term motor memory. J. Neurosci..

[48] Fischer S., Hallschmid M., Elsner A.L., Born J. (2002). Sleep forms memory for finger skills. Proc. Natl. Acad. Sci. USA.

[49] Walker M.P., Brakefield T., Morgan A., Hobson J.A., Stickgold R. (2002). Practice with sleep makes perfect: sleep-dependent motor skill learning. Neuron.

[50] Zhu J.P., Zhang J.Y. (2025). Brief memory reactivation may not improve visual perception. Vision Res..

[51] Chen C.Y., Op de Beeck H. (2021). Perceptual learning with complex objects: a comparison between full-practice training and memory reactivation. eNeuro.

[52] Kehrer L. (1989). Central performance drop on perceptual segregation tasks. Spat. Vis..

[53] Gurnsey R., Di Lenardo D., Potechin C. (2004). Backward masking and the central performance drop. Vision Res..

[54] Morikawa K. (2000). Central performance drop in texture segmentation: The role of spatial and temporal factors. Vision Res..

[55] Erdfelder E., Faul F., Buchner A. (1996). GPOWER: A general power analysis program. Behav. Res. Methods Instrum. Comput..

[56] Wichmann F.A., Hill N.J. (2001). The psychometric function: I. Fitting, sampling, and goodness of fit. Percept. Psychophys..

[57] Van Rossum G., Drake F.L. (2009). Python 3 Reference Manual. CreateSpace.

[58] JASP Team. (2025). JASP (Version 0.19.3) [Computer software]. https://jasp-stats.org.

[59] The MathWorks Inc. (2024). MATLAB version R2024a (24.1.0). https://mathworks.com.

[60] Jeffreys H. (1961).

[61] Rouder J.N., Morey R.D., Speckman P.L., Province J.M. (2012). Default Bayes factors for ANOVA designs. J. Math. Psychol..

[62] van den Bergh D., van Doorn J., Marsman M., Draws T., van Kesteren E.J., Wagenmakers E.J. (2019). A tutorial on conducting and interpreting a Bayesian ANOVA in JASP. LAnnee Psychologique.

