# Supplementary material for: No practice and still perfect: Different routes for promoting offline gains in perceptual learning
Source: iScience. 2026 Oct 13;29(11):117839. doi: 10.1016/j.isci.2026.117839 (PMC13683855; doi:10.1016/j.isci.2026.117839)
Supplement: Supplementary file 1 [file mmc1.pdf]

iScience, Volume *29*

Supplemental information

No practice and still perfect: Different routes for promoting offline gains in perceptual learning

Ylka Kolken, Mark J. Roberts, Parisa Naseri, Federico De Martino, Nitzan Censor, and Peter De Weerd

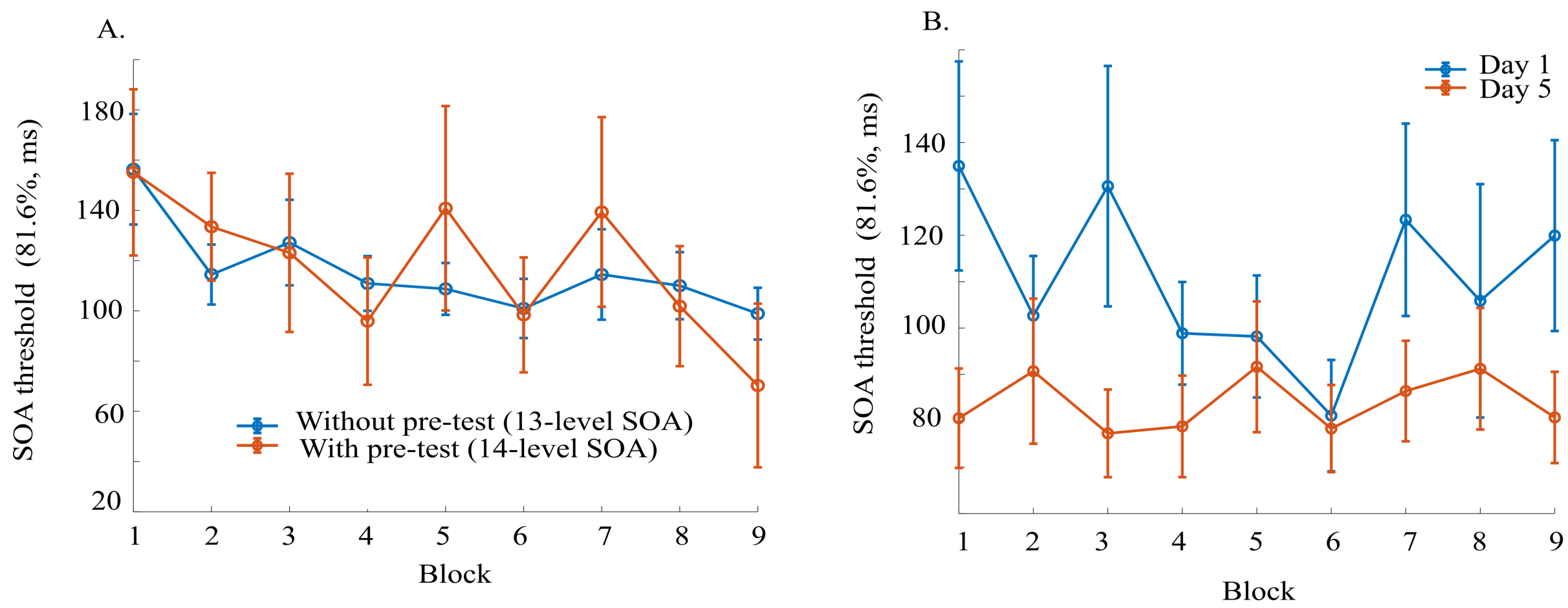

Figure S1: Related to Figure 3. A. Similar learning curves for participants trained with the 14-level SOA range and pre-training compared to participants trained with the 13-level SOA range without pre-training ($BF_{10} = 0.056$). Data represent mean ± SEM. B. Learning curves of the participants in the *control* condition showing between-session learning, rather than within-session learning. An LMM revealed a significant main effect of Day (F(1,175)=7.90, p<.001), but no main effect of Block (F(1,175)=0.62, p=.43) and no Day × Block interaction (F(1,175)=0.53, p=.467), indicating an overall improvement between days without evidence of systematic within-session learning. Data represent mean ± SEM.

Table S1: Related to Figure 5. Division of participants over the experiments. The old protocol used the 14-level SOA range and included a pre-training procedure, whereas the new protocol used the 13-level SOA range and omitted pre-training.

Experiment	N included	N excluded
Dual-task TDT	30	15
Single-task TDT with the same stimulus	10	0
Single-task TDT with modified stimulus and old protocol	15	8
Single-task TDT with modified stimulus and new protocol	23	2

Table S2: Mixed Linear Model (dual-task TDT, related to Figure 1). Reference condition = Control, condition 2 = *full training*, condition 3 = *reminder* baseline = day 1.

Predictor	β	SE	z	p
Intercept	188.77	28.79	6.56	< .001
Time	-5.84	22.11	-0.26	.792
condition 2	-12.95	40.71	-0.32	.750
condition 3	-16.90	40.71	-0.42	.678
Time × condition 2	-77.12	31.27	-2.47	.014
Time × condition 3	-67.83	31.27	-2.17	.030

Table S3: Related to Figure 2. Overview of behavioral paradigms and parameters in related studies, referred to by the first author and publication year (full references in the reference list). Under Method, we refer to random (all SOA levels are presented intermixed) or fixed (blocks of trials are separated per SOA level) stimulus presentation modes. For Censor and Sagi (2008) thresholds, we used the sample that received 26 trials per block (their Fig. 2). For Harris and Sagi (2018) we used the 4-degree eccentricity condition (their Fig. 2). For Meng et al., 2014 we used only the non-dyslexic participants (children). Some values were extracted using PlotDigitizer (https://plotdigitizer.com), so numbers are approximate.

**Paper**	**Sample**	**Target**	**Method**	**Eccentricity**	**Trials/soa**	**Trials**	**Feedback**	**Threshold criterion**
Dual-task TDT	30	17	Random	$5.72°$	18	252	Fixation	81.6
Preliminary experiment	10	17	Random	$5.72°$	18	252	No	81.6
Amar-Halpert, 2017	24	10	Random	$5.72°$	18	252	Fixation	81.6
Berard, 2015	9	13	Fixed	$5-9°$	39	273	Fixation	75
Yotsumoto, 2009	6	13	Fixed	$5-9°$	26	273	Fixation	75
Censor, 2006	7	40	Fixed	$4.46-6°$	26	104	Fixation	81.6
Karni, 1991	6	10	Fixed	$2.5-5°$	50	$\sim$1000	Fixation	80
Harris, 2012	55	10	Random	$5.3°$	18	252	Not reported	Not reported
Harris, 2018	5	10	Random	$4°$	18	252	Fixation	Not reported
Kondat, 2023	62	10	Random	$5.46°$	18	252	Fixation	81.6
Kondat, 2024	41	10	Random	$5.46°$	18	252	Fixation	81.6
Meng, 2014	18	36	Staircase	$2.5-5°$	n/a	180	No	66.7
Qu, 2014	24	17	Fixed	$13-19°$	16	144	No	81.6
Wang, 2013	8	13	Staircase	$4-6.5°$	n/a	80	Fixation	79.4

Table S4: Mixed linear model (single-task TDT, related to Figure 3) with the interaction effect. Reference condition = Control, condition 2 = *full training*, condition 3 = *reminder*, baseline = day 1.

Predictor	β	SE	z	p
Intercept	123.675	17.294	7.151	$<$ .001
Time	-35.983	14.539	-2.475	.013
condition 2	-4.730	24.458	0.193	.847
condition 3	2.683	24.458	0.110	.913
Time $\times$ condition 2	-13.061	20.561	-0.635	.525
Time $\times$ condition 3	-67.83	20.561	-0.397	.691

Table S5: Mixed linear model (single-task TDT, related to Figure 3) without the interaction effect. Reference condition = Control, baseline = day 1.

Predictor	β	SE	z	p
Intercept	130.061	13.466	9.659	$<$ .001
Time	-43.060	8.161	-5.276	$<$.001
condition 2	-7.056	15.949	-0.442	.658
condition 3	-4.689	15.940	-0.294	.769

Supplementary References

Amar-Halpert, R., Laor-Maayany, R., Nemni, S., Rosenblatt, J. D., & Censor, N. (2017). Memory reactivation improves visual perception. *Nat Neurosci*, *20*(10), 1325–1328. https://doi.org/10.1038/nn.4629

Berard, A. V., Cain, M. S., Watanabe, T., & Sasaki, Y. (2015). Frequent video game players resist perceptual interference. *PLOS ONE*, *10*(3), e0120011. https://doi.org/10.1371/journal.pone.0120011

Censor, N., & Sagi, D. (2008). Benefits of efficient consolidation: Short training enables long-term resistance to perceptual adaptation induced by intensive testing. *Vision Res*, *48*(7), 970–7. https://doi.org/10.1016/j.visres.2008.01.016

Harris, H., Gliksberg, M., & Sagi, D. (2012). Generalized perceptual learning in the absence of sensory adaptation. *Curr Biol*, *22*(19), 1813–7. https://doi.org/10.1016/j.cub.2012.07.059

Harris, H., & Sagi, D. (2018). Visual learning with reduced adaptation is eccentricity-specific. *Scientific Reports*, *8*(1), 608. https://doi.org/10.1038/s41598-017-18659-9

Karni, A., & Sagi, D. (1991). Where practice makes perfect in texture discrimination: Evidence for primary visual cortex plasticity. *Proc Natl Acad Sci U S A*, *88*(11), 4966–70. https://doi.org/10.1073/pnas.88.11.4966

Kondat, T., Aderka, M., & Censor, N. (2023). Modulating temporal dynamics of performance across retinotopic locations enhances the generalization of perceptual learning. *iScience*, *26*(11), 108276. https://doi.org/10.1016/j.isci.2023.108276

Kondat, T., Tik, N., Sharon, H., Tavor, I., & Censor, N. (2024). Distinct neural plasticity enhancing visual perception [Featured Article, Research Articles, Behavioral/Cognitive]. *Journal of Neuroscience*, *44*(36), e0301242024. https://doi.org/10.1523/JNEUROSCI.0301-24.2024

Meng, X., Lin, O., Wang, F., Jiang, Y., & Song, Y. (2014). Reading performance is enhanced by visual texture discrimination training in chinese-speaking children with developmental dyslexia. *PLoS ONE*. https://doi.org/10.1371/journal.pone.0108274

Qu, Z., Wang, Y., Zhen, Y., Hu, L., Song, Y., & Ding, Y. (2014). Brain mechanisms underlying behavioral specificity and generalization of short-term texture discrimination learning. *Vision Research*, *105*, 107–116. https://doi.org/10.1016/j.visres.2014.10.010

Wang, R., Cong, L.-J., & Yu, C. (2013). The classical tdt perceptual learning is mostly temporal learning. *Journal of Vision*, *13*(5), 1–9. https://doi.org/10.1167/13.5.9

Yotsumoto, Y., Watanabe, T., & Sasaki, Y. (2008). Different dynamics of performance and brain activation in the time course of perceptual learning. *Neuron*, *57*(6), 827–33. https://doi.org/10.1016/j.neuron.2008.02.034
